# Negative Correlation Between Secreted Phosphoprotein 1 and the Treg/Th17 Ratio in Non-Valvular Atrial Fibrillation

**DOI:** 10.31083/RCM44147

**Published:** 2025-10-28

**Authors:** Chao-Jun Yang, Bo Fu, Yi-Fan Huang, Jing-Yi Wu, Zhi-Xing Fan, Ya-Hui Li

**Affiliations:** ^1^Department of Cardiology, First Clinical Medical College, China Three Gorges University, 443002 Yichang, Hubei, China; ^2^Hubei Key Laboratory of Ischemic Cardiovascular Disease, 443003 Yichang, Hubei, China; ^3^Hubei Provincial Clinical Research Center for Ischemic Cardiovascular Disease, 443003 Yichang, Hubei, China; ^4^Department of Traditional Chinese Medicine, Xianning Central Hospital, The First Affiliated Hospital of Hubei University of Science and Technology, 437000 Xianning, Hubei, China; ^5^Division of Cardiology, Department of Internal Medicine, Tongji Hospital, Tongji Medical College, Huazhong University of Science and Technology, 430030 Wuhan, Hubei, China

**Keywords:** secreted phosphoprotein 1, Treg/Th17, non-valvular atrial fibrillation, inflammation, fibrosis

## Abstract

**Background:**

Atrial fibrillation (AF) is a common cardiac arrhythmia strongly associated with an imbalance between T helper 17 (Th17) cells and regulatory T cells (Treg). Secreted phosphoprotein 1 (SPP1), an immune signaling molecule implicated in AF pathogenesis, may shift the Th17/Treg cell balance in non-valvular AF (NVAF). This study aimed to explore the regulatory effects of SPP1 on the balance of Th17 and Treg cells in NVAF.

**Methods:**

Venous blood samples were collected from 58 patients with NVAF (observation group) and 58 age- and sex- matched healthy controls (control group). The serum concentrations of SPP1, along with the percentages of Treg and Th17 cells, and the levels of their associated cytokines, were measured. Correlation analysis was employed to evaluate the association between serum SPP1 levels and the Treg/Th17 cell ratio. In parallel, an experimental rat model of AF was established to investigate the expression of SPP1, related inflammatory factors, and fibrin within the left atrial tissue.

**Results:**

NVAF patients showed significantly higher serum levels of SPP1 and certain inflammatory cytokines (interleukin (IL)-17A and IL-23) than the controls. NVAF patients exhibited increased Th17 cells and elevated collagen I levels. Meanwhile, Treg cell frequency and IL-10 levels were significantly reduced compared to controls. Consequently, the Treg/Th17 ratio was significantly lower in NVAF patients. Notably, a significant inverse correlation was identified between serum SPP1 concentrations and the Treg/Th17 ratio. Consistent results were also obtained in animal models of AF, further supporting these findings.

**Conclusion:**

Our findings suggest that elevated SPP1 levels disrupt the Treg/Th17 cell balance in NVAF patients, promoting inflammation and fibrosis. These findings indicate that SPP1 represents a promising therapeutic target for the prevention and management of NVAF.

## 1. Introduction

Non-valvular atrial fibrillation (NVAF) is a common tachyarrhythmia strongly associated with myocardial fibrosis driven by chronic inflammation [1]. Although atrial fibrillation (AF) has traditionally been regarded as a cardiac rhythm disorder resulting from atrial myocyte remodeling, emerging evidence increasingly implicates immune dysregulation in its pathogenesis [2, 3]. Regulatory T cells (Treg) and T helper 17 cells (Th17) are key CD4^+^ T cells subsets that differentiate from naive CD4^+^ T cells and critically regulate cellular immunity [4]. As Treg cells suppress immunity while Th17 cells promote inflammation, the Treg/Th17 ratio serves as a biomarker of inflammatory status and a predictor of diseases such as atherosclerosis [5, 6]. Given its chronic inflammatory nature, AF involves complex interactions among various immune cells and inflammatory cytokines [7, 8]. Disruption of circulating Treg/Th17 homeostasis has also been reported in patients with AF and rheumatoid arthritis [9]. Moreover, a decreased Treg/Th17 ratio reflects a pro-inflammatory state and predicts an increased risk of AF following off-pump coronary artery bypass grafting [10]. Secreted phosphoprotein 1 (SPP1), also known as osteopontin, is an extracellular matrix protein implicated in various pathological processes, including calcification, fibrosis, and inflammation [11, 12, 13]. Recent research has underscored the potential involvement of SPP1 in the pathogenesis of AF. SPP1 in atrial fibroblasts has been reported to promote atrial fibrosis through the Akt/glycogen synthase kinase-3 (GSK)-3β/β-catenin and autophagy-related pathways [14]. Additionally, bioinformatic analyses and single-cell transcriptomes have shown that SPP1 promotes macrophage expansion and mediates cross-talk between atrial immune and stromal cells in AF [15]. Meanwhile, emerging evidence suggests that SPP1 modulates immune responses by promoting Th17 differentiation and inhibiting Treg function, thereby contributing to AF initiation [16, 17]. Therefore, we hypothesize that SPP1 may be inversely correlated with the Treg/Th17 ratio in NVAF patients, thereby contributing to pro-inflammatory conditions and enhancing the susceptibility to cardiac dysrhythmia. The objective of this study was to investigate the association between SPP1 and peripheral Treg/Th17 homeostasis by examining serum SPP1 levels and the Treg/Th17 ratio, aiming to enhance our understanding of immune-related AF pathogenesis and identify novel targets for AF therapy.

## 2. Materials and Methods

### 2.1 Study Design and Patients

Fifty-eight patients with non-valvular AF treated at the first clinical medical college of China Three Gorges University and Tongji Hospital between January 2023 and December 2024 were included in the observation group. The inclusion criteria were as follows: (1) electrocardiograph (ECG) showing absence of P waves, f-waves at 350–600 bpm, and irregularly irregular QRS complexes, as defined by current guidelines [18]; and (2) documented AF duration >3 months. Exclusion criteria were as follows: (1) history of valvular heart disease; (2) rheumatic heart disease or dilated cardiomyopathy; (3) hyperthyroidism; (4) malignancy; and (5) any surgery within the preceding six months. The control group comprised 58 hospitalized patients without a history of AF, matched for age and other baseline characteristics. No significant differences were observed in age, hypertension, sex, coronary artery disease, smoking history, fasting blood glucose, serum creatinine, total cholesterol, triglycerides, high-density lipoprotein, and low-density lipoprotein (*p* > 0.05). The observation group had a significantly lower ejection fraction than the control group (*p* < 0.05). Additionally, both the left atrial diameter and the left ventricular end-diastolic diameter were significantly larger in the observation group than in the control group (*p* < 0.05). All patients were thoroughly informed about the study’s objectives and procedures and provided written informed consent. This study received approval from the Medical Ethics Committee of Tongji Hospital.

### 2.2 Construction of AF Rat Model

Sixteen specific pathogen-free (SPF) Sprague-Dawley (SD) rats, aged 6 to 8 weeks and weighing between 200 and 220 grams, were supplied by the Animal Experiment Center of Three Gorges University. An AF animal model was established in eight SD rats through daily intravenous injection of a mixture of acetylcholine and calcium chloride (ACH-CaCl_2_, 1 mg⋅kg^-1^ day^-1^) via the tail vein for four consecutive weeks (AF group). The remaining eight rats were raised under normal conditions and served as negative controls (the normal group). After discontinuation of injections, surface ECGs were recorded in conscious rats. Successful induction of the AF model was indicated by the absence of the P wave, supplanted by small f-waves with a frequency of 350–600 beats/min on the ECG. All rats were anesthetized using 3% sodium pentobarbital, administered intraperitoneally at 30 mg/kg. After euthanasia by air embolism, atrial tissues were collected for subsequent analysis. The reporting of animal experiments followed the Animal Research: Reporting of In Vivo Experiments (ARRIVE) guidelines. All animal experiments received authorization from the institutional ethics committees of China Three Gorges University (2024050L).

### 2.3 Isolation of Peripheral Blood Mononuclear Cells (PBMCs)

Five milliliters of fasting venous blood were collected from each patient and processed using density gradient centrifugation. At room temperature, the blood was combined with an equal volume of pre-warmed phosphate-buffered saline (PBS) and lymphocyte separation medium in a 50 mL centrifuge tube, followed by centrifugation at 1700 rpm for 15 minutes. After centrifugation, the sample was separated into four distinct layers. The mononuclear cell layer was meticulously transferred to a 15 mL tube and subsequently washed with PBS, using a volume 2–5 times greater than that of the cell layer, and centrifuged again (1000 rpm, 10 min, room temperature). After discarding the supernatant, the cells were resuspended in PBS to achieve a PBMC concentration of 2 × 10^6^/mL.

### 2.4 Flow Cytometry Detection of Treg and Th17 Cell Proportions in PBMCs

#### 2.4.1 Detection of Treg Cell Proportion

For this analysis, microcentrifuge tubes for antibody incubation were divided into five groups: one for blank control, three for single-staining of T cells (CD4^+^, CD25^+^, and CD127^–^, respectively), and one for triple-staining of Treg cells (CD4^+^CD25^+^CD127^–^). The antibodies employed for subsequent staining included anti-human CD4-FITC (Ebioscience, RRID: AB_1272074, San Diego, CA, USA), anti-human CD25-PE (Ebiosciences, RRID: AB_2744720, San Diego, CA, USA), and anti-human CD127-PE-CY7 (Ebiosciences, RRID: AB_2043801, San Diego, CA, USA). Subsequently, 5 µL of the appropriate antibodies were added to the tubes for each group, with a mixture of the three antibodies used for the Treg cell triple-staining tube. Each tube contained 100 µL of cell suspension (isolated PBMCs). The mixture was incubated at 4 °C in the dark for 30 minutes, then ice-cold PBS (4 °C) was added, and the suspension was centrifuged at 1500 rpm for 5 minutes to form a cell pellet. Following the removal of the supernatant, the cells were washed twice with 2 mL of ice-cold PBS and resuspended in 300 µL PBS. The prepared samples were then analyzed using a flow cytometer (Beckman Kurt Technology, model: CytoFLEX, Brea, CA, USA).

#### 2.4.2 Detection of Th17 Cell Proportion

The isolated PBMCs were placed in 24-well plates and stimulated for 6 h at 37 °C with 5% CO_2_ (ESCO, CLM-1708-8-NF, Singapore) in the presence of a stimulation cocktail containing PMA (25 ng/mL, Sigma, CAS No.: 108-65-6, St. Louis, MO, USA), ionomycin (1 µg/mL, Sigma, CAS No.: 56092-82-1, St. Louis, MO, USA), monensin (1.4 µg/mL, MCE, CAS No.: 22373-78-0, Monmouth Junction, NJ, USA), and brefeldin A (3 µg/mL, MCE, CAS No.: 20350-15-6, Monmouth Junction, NJ, USA). Subsequently, the PBMCs were collected, washed with PBS at 37 °C, centrifuged at 1500 rpm for 5 minutes at room temperature, and the supernatant was discarded. The cells were then resuspended in PBS to achieve a concentration of 2 × 10^6^/mL. For surface staining, 100 µL of the cell suspension was added to tubes labeled as blank, CD3^+^ T cell, CD8^–^ T cell, interleukin (IL)-17^+^ T cell, and CD3^+^CD8^–^IL-17^+^ Th17 cell. Each tube received 5 µL of the corresponding surface antibodies, including anti-human CD3 FITC antibody (Ebioscience, RRID: AB_2572431, San Diego, CA, USA), anti-human CD8 APC antibody (Ebioscience, RRID: AB_10669564, San Diego, CA, USA), and anti-human IL-17A-PE antibody (Ebioscience, RRID: AB_11063994, San Diego, CA, USA), with a mixture of the three antibodies added to the CD3^+^CD8^–^IL-17^+^ Th17 cell tubes. After thorough mixing, the samples were incubated at 4 °C in the dark for a duration of 30 minutes, then washed twice with 2 mL of pre-chilled PBS, and centrifuged. After discarding the supernatant, 100 µL of Fix buffer was added for fixation under the same conditions. Cells were then permeabilized with two washes of 1× Perm buffer, followed by centrifugation. For intracellular staining, 100 µL of 1× Perm buffer containing 5 µL of anti-human IL-17-PE antibody was added to the IL-17^+^ and CD3^+^CD8^–^IL-17^+^ tubes. The samples underwent another incubation at 4 °C in darkness for an hour, followed by two washes with PBS. They were then centrifuged at 1700 rpm for 5 minutes and finally resuspended in 300 µL of PBS for flow cytometric analysis.

#### 2.4.3 RT‒PCR Detection of Forkhead Box Protein P3 (Foxp3) and Retinoic Acid-related Orphan Nuclear Receptor C (RORC)

The transcription factor *Foxp3* is specifically involved in the proliferation and differentiation of regulatory T cells, whereas *RORC* functions as a key transcription factor for Th17 cells. Total RNA of PBMCs was extracted utilizing the TRIzol method. Subsequently, reverse transcription of RNA to cDNA was performed using a cDNA synthesis kit (Sigma, CAS No.: 11483188001, USA), followed by RT-PCR using the SYBR premix Kit (Takara company, Kusatsu, Shiga, Japan). PCR conditions were as follows: 95 °C for 2 minutes, 95 °C for 15 seconds → 60 °C for 30 seconds → 72 °C for 30 seconds (45 cycles). The 2^–Δ⁢Δ⁢Ct^ method was used to calculate relative mRNA expression changes. The mRNA levels were normalized to GAPDH. The following primers were used: *Foxp3* forward, 5^′^-AACAGCACATTCCCAGAGTTCC-3^′^ and reverse, 5^′^-CATTGAGTGTCCGCTGCTTC-3^′^; *RORC* forward, 5^′^-CCGAGGATGAGATTGCCCTCT-3^′^ and reverse, 5^′^-GGTGGCAGCTTTGCCAGGAT-3^′^; *GAPDH* forward 5^′^-CCACATCGCTCAGACACCAT-3^′^ and reverse, 5^′^-CCAGGCGCCCAATACG-3^′^. The primers for PCR amplification were obtained from Shanghai Shenggong Biotechnology.

### 2.5 ELISA Detection of Serum Protein Expression

Blood samples were collected in standard tubes, allowed to clot at room temperature for 30 minutes and then centrifuged at 3000 rpm for 20 minutes. The resulting supernatant serum was then separated and stored at –80 °C until further analysis. The levels of SPP1 (Shanghai Jianglai Biotechnology Co., Ltd., Cat. No.: JL10368, Shanghai, China), collagen I (Shanghai Jianglai Biotechnology Co., Ltd., Cat. No.: JL47278, Shanghai, China), IL-10 (Xinboseng Biotechnology Co., Ltd., Cat. No.: EHC009.96, Shanghai, China), IL-17A (Xinboseng Biotechnology Co., Ltd., Cat. No.: EHC170.96, Shanghai, China), and IL-23 (Xinboseng Biotechnology Co., Ltd., Cat. No.: EHC171.96, Wuhan, China) were measured following the manufacturer’s ELISA kit instructions.

### 2.6 ELISA Detection of Protein Expression in Rats’ Left Atrium

The levels of SPP1 (Shanghai Jianglai Biotechnology Co., Ltd., Cat. No.: JL14517, Shanghai, China), collagen I (Wuhan Jilide Biotechnology Co., Ltd., Cat. No.: J23744, Wuhan, China), IL-10 (Wuhan Jilide Biotechnology Co., Ltd., Cat. No.: J22449, Wuhan, China), IL-17A (Wuhan Jilide Biotechnology Co., Ltd., Cat. No.: J23469, Wuhan, China), and IL-23 (Wuhan Jilide Biotechnology Co., Ltd., Cat. No.: J22374, Wuhan, China) in the left atrium of rats was detected using ELISA following the manufacturer’s guidelines.

### 2.7 Histological Analysis

The left atrial tissue samples were embedded in paraffin and sectioned at 5 µm thickness. Masson’s trichrome staining was employed to assess the extent of fibrosis in the left atrium. These sections were examined under a microscope, and the measurements were taken using Image Pro-Plus software (Media Cybernetics Inc., Silver Spring, MD, USA).

### 2.8 Biochemical Index Detection

HDL-C, LDL-C, TC, TG and FBG were measured by the clinical laboratory department of Tongji Hospital.

### 2.9 Statistical Analysis

Statistical analyses were conducted utilizing SPSS version 22.0 (IBM Corp., Armonk, NY, USA). Continuous variables are expressed as mean ± standard deviation. Homogeneity-of-variance testing was performed first. If variances were equal (*p* > 0.05), the two-sample *t*-test was used for inter-group comparisons; if variances were unequal (*p* < 0.05), Welch’s *t*-test was applied. Categorical data are represented as n (%), with the chi-square test applied. Pearson’s correlation analysis was performed to assess correlations, with a significance threshold set at *p* < 0.05.

## 3. Results

### 3.1 Baseline Characteristics in Two Groups

Baseline characteristics showed no significant differences between the observation and control groups in terms of age, sex, hypertension, coronary artery disease, smoking status, fasting glucose, serum creatinine, or lipid profile parameters (*p* > 0.05). In contrast, the observation group exhibited a significantly lower ejection fraction and larger left atrial and left ventricular end-diastolic diameters than the control group (*p* < 0.05) (Table 1).

**Table 1.** **Baseline characteristics in two groups**.

	Observation group (n = 58)	Control group (n = 58)	*t*/χ^2^	*p*
Gender, Male (%)	34 (58.6)	37 (63.8)	0.327	0.568
Age (years)	67.8 ± 7.6	66.4 ± 6.2	1.087	0.279
Hypertension, n (%)	42 (72.4)	38 (65.5)	0.644	0.422
Coronary heart disease, n (%)	35 (60.3)	37 (63.8)	0.146	0.702
Ejection fraction (%)	47.35 ± 9.14	60.13 ± 7.44	8.259	<0.001
Left atrial diameter (mm)	40.13 ± 6.41	32.52 ± 3.98	7.681	<0.001
Left ventricular end diastolic diameter (mm)	44.23 ± 4.98	32.52 ± 4.61	13.141	<0.001
Smoking history, n (%)	19 (32.8)	21 (36.2)	0.153	0.696
Fasting blood glucose (mmol/L)	4.5 ± 0.3	4.6 ± 0.4	–1.523	0.130
Serum creatinine (umol/L)	76.1 ± 15.4	79.5 ± 16.1	1.162	0.248
Total cholesterol (mmol/L)	4.2 ± 1.1	4.1 ± 1.0	0.512	0.609
Triglycerides (mmol/L)	1.6 ± 0.7	1.5 ± 0.8	0.716	0.475
High-density lipoprotein (mmol/L)	1.3 ± 0.3	1.4 ± 0.4	–1.523	0.130
Low-density lipoprotein (mmol/L)	2.3 ± 0.7	2.2 ± 0.8	0.716	0.475

### 3.2 Proportions of Treg and Th17 Cells in PBMCs of the Two Groups

The proportions of CD4^+^CD25^+^CD127^–^ Treg cells and CD3^+^CD8^–^IL17^+^ Th17 cells in PBMCs were determined. Compared to the control group, the observation group exhibited a significant reduction in the proportion of CD4^+^CD25^+^CD127^–^ Treg cells (*p* < 0.001). Conversely, there was a significant increase in the proportion of CD3^+^CD8^–^IL17^+^ Th17 cells (*p* < 0.001), and the Treg/Th17 ratio was significantly decreased (*p* < 0.001) (Table 2).

**Table 2.** **Proportions of Treg and Th17 cells in PBMCs of the two groups ($\bar{x}$ ± *s*)**.

	Control group (n = 58)	Observation group (n = 58)	*t*	*p*
CD4^+^CD25^+^CD127^–^ Treg (%)	5.9 ± 0.9	3.9 ± 0.7^*^	13.359	<0.001
CD3^+^CD8^–^IL17^+^ Th17 (%)	1.1 ± 0.4	2.4 ± 0.6^*^	–13.730	<0.001
Treg/Th17	5.3 ± 0.5	1.6 ± 0.2^*^	52.326	<0.001

*p < 0.001 compared with the control group. Treg, regulatory T cells; Th17, T helper 17; PBMCs, peripheral blood mononuclear cells.

### 3.3 RT‒PCR Detection of Foxp3 and RORC mRNA Levels

**Fig. 1.**
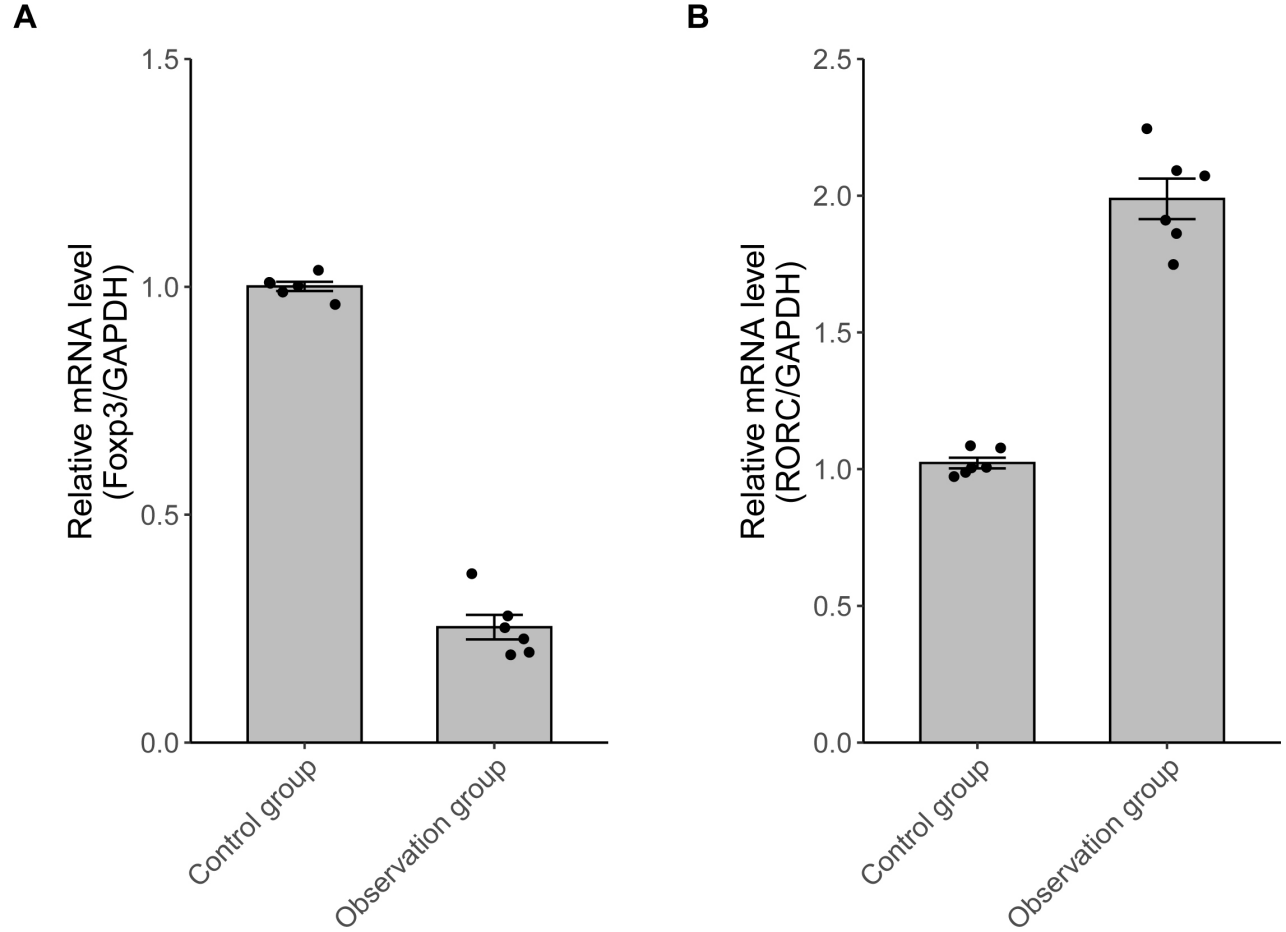
**The *Foxp3* and *RORC* mRNA expressions**. (A) In the observation group, *Foxp3* mRNA was lower (n = 6, *p* < 0.05); (B) *RORC* mRNA was higher (n = 6, *p* < 0.05). Foxp3, forkhead box protein P3; RORC, retinoic acid-related orphan nuclear receptor C; GAPDH, glyceraldehyde-3-phosphate dehydrogenase.

In the observation group, *RORC* mRNA was significantly up-regulated, whereas *Foxp3* mRNA was down-regulated (*p* < 0.05) (Fig. 1).

### 3.4 ELISA Detection of Serum SPP1, Collagen I, IL-10, IL-17A, and IL-23 Levels

Serum levels of SPP1, collagen I, IL-10, IL-17A, and IL-23 were measured using ELISA. Compared to the control group, the observation group exhibited a significant increase in SPP1 and collagen I concentrations (*p* < 0.001). Pro-inflammatory cytokines associated with Th17 cells (IL-17A and IL-23) were significantly increased, whereas the anti-inflammatory cytokines linked to Treg cells (IL-10) were significantly reduced (*p* < 0.001) (Table 3).

**Table 3.** **Levels of SPP1, Collagen I, IL-10, IL-17A, and IL-23 in patients ($\bar{x}$ ± *s*)**.

	Control group (n = 58)	Observation group (n = 58)	*t*	*p*
SPP1 (ng/mL)	6.7 ± 1.2	16.3 ± 3.4^*^	–20.277	<0.001
Collagen I (ug/mL)	1.1 ± 0.2	1.9 ± 0.3^*^	16.898	<0.001
IL-10 (pg/mL)	421.3 ± 33.2	199.6 ± 21.5^*^	42.687	<0.001
IL-17A (pg/mL)	17.4 ± 2.9	49.8 ± 7.8^*^	–29.652	<0.001
IL-23 (pg/mL)	37.6 ± 4.3	77.5 ± 8.1^*^	–33.135	<0.001

*p < 0.001 compared with the control group. SPP1, secreted phosphoprotein 1; IL, interleukin.

### 3.5 ELISA Detection of Left Atrium SPP1, IL-10, IL-17A, and IL-23 Levels

**Fig. 2.**
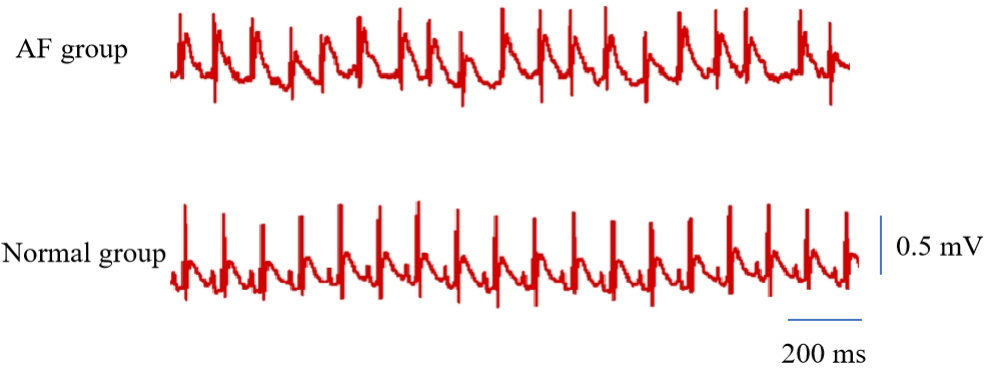
**A typical ECG of AF**. AF, atrial fibrillation; ECG, electrocardiograph.

After tail-vein injection, all eight rats exhibited typical AF waveforms: f waves replaced P waves and R-R intervals were irregular. The duration of AF in all rats exceeded 10 seconds (18.92 ± 4.76 s, n = 8). Fig. 2 shows a typical ECG of AF. Left atrial tissue concentrations of SPP1, IL-10, IL-17A, and IL-23 were quantified using ELISA. Compared to the normal group, the atrial fibrillation group showed significantly elevated levels of SPP1 (*p* < 0.001). Pro-inflammatory cytokines associated with Th17 cells (IL-17A and IL-23) were significantly increased, whereas the anti-inflammatory cytokines linked to Treg cells (IL-10) were significantly reduced (*p* < 0.001) (Table 4).

**Table 4.** **Levels of SPP1, IL-10, IL-17A, and IL-23 in rats left atrium ($\bar{x}$ ± *s*)**.

	Normal group (n = 8)	Atrial fibrillation group (n = 8)	*t*	*p*
SPP1 (pg/mgprot)	2.4 ± 0.3	9.1 ± 1.2^*^	–15.321	<0.001
IL-10 (pg/mgprot)	64.7 ± 3.4	32.8 ± 2.9^*^	20.190	<0.001
IL-17A (pg/mgprot)	57.7 ± 5.5	103.4 ± 6.1^*^	15.738	<0.001
IL-23 (pg/mgprot)	26.4 ± 3.2	49.5 ± 3.7^*^	13.356	<0.001

*p < 0.001 compared with the normal group. SPP1, secreted phosphoprotein 1; IL, interleukin.

### 3.6 Detection of Atrial Fibrosis

**Fig. 3.**
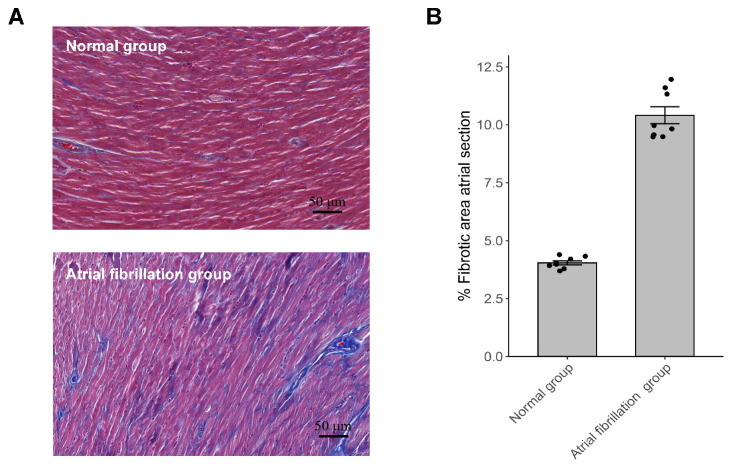
**Atrial fibrosis was assessed by Masson staining**. (A) Representative images of Masson staining of the left atrium (magnification, ×200); (B) Quantitative analysis of atrial fibrosis (%) calculated from Masson staining (n = 8, *p* < 0.001).

Atrial fibrosis was assessed using Masson’s trichrome staining. Our findings indicate that left atrial fibrosis was significantly more extensive in the AF group than that of the control group (Fig. 3).

### 3.7 Correlation Analysis Between SPP1 and Treg/Th17

**Fig. 4.**
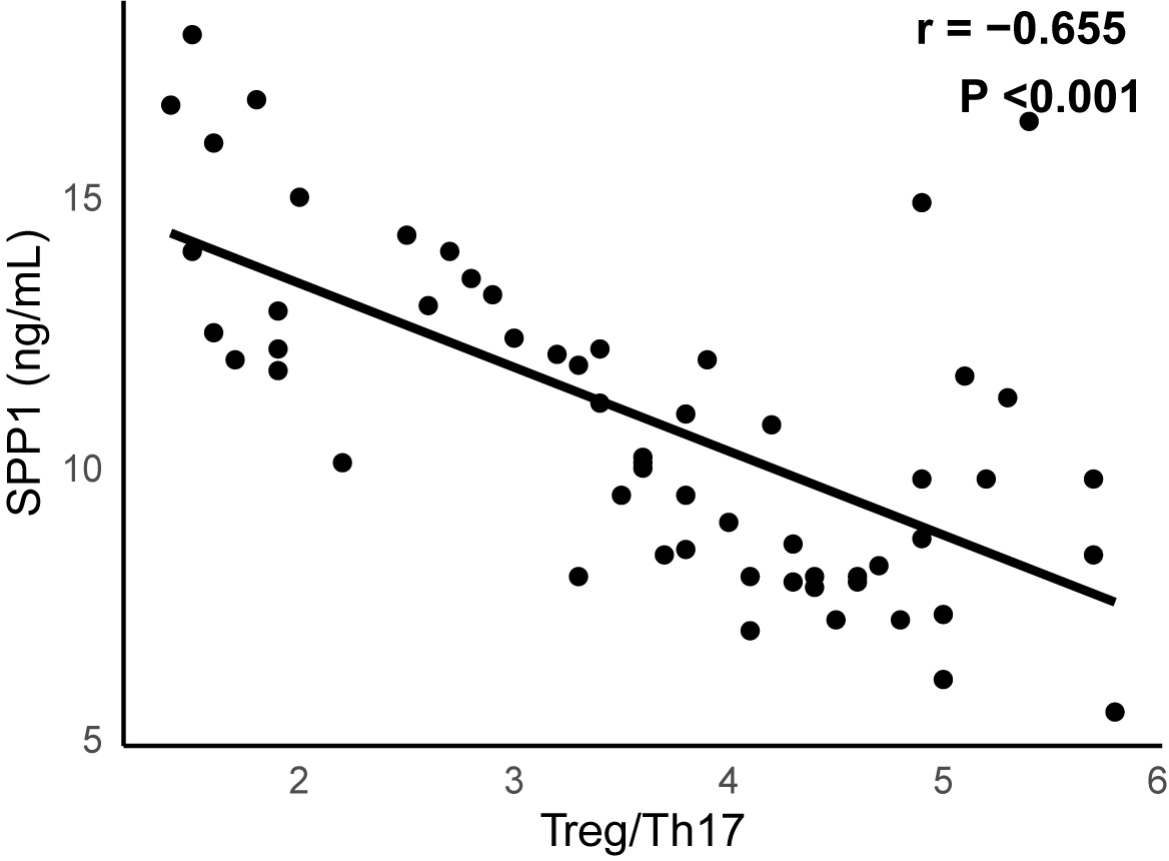
**A significant negative correlation between SPP1 and Treg/Th17 (n = 58, *r* = –0.655, *p* < 0.001)**. SPP1, secreted phosphoprotein 1; Treg, regulatory T cells; Th17, T helper 17.

Correlation analysis revealed a statistically significant inverse relationship between SPP1 and the Treg/Th17 ratio (n = 58, *r* = –0.655, *p* < 0.001) (Fig. 4).

## 4. Discussion

SPP1, also known as osteopontin, has gained recognition for its role in immune modulation and fibrotic remodeling in various cardiovascular diseases [19, 20]. In this study, we identified significantly elevated serum SPP1 levels in patients with NVAF, accompanied by a marked reduction in Treg cells and an increase in Th17 cells. As a result, the Treg/Th17 ratio was markedly reduced in NVAF patients. These immunological changes correlated with elevated pro-inflammatory cytokines (IL-17A and IL-23) and reduced anti-inflammatory markers (IL-10). Similar findings were observed in animal models of AF, further supporting these observations.

Furthermore, we observed a strong inverse correlation between serum SPP1 levels and the Treg/Th17 ratio, suggesting that SPP1 may exert regulatory effects on immune cell differentiation in patients with NVAF. A similar Treg/Th17 imbalance has been reported in patients with inflammatory lung injury [21]. However, Chen *et al*. [22] observed elevated SPP1 levels along with an increased Th17/Treg ratio and, unexpectedly, a concomitant rise in Treg percentage. Given the evidence of reduced Treg cells in AF patients, this discrepancy may be attributed to differences in study populations, or disease stages.

The fibrotic and inflammatory roles of SPP1 have been well-documented in various cardiovascular contexts. Recent research indicates that SPP1 may facilitate atrial fibrosis through activation of the Akt/GSK-3β/β-catenin signaling pathway, disruption of autophagy, and enhanced extracellular matrix accumulation [14]. This aligns with our findings of increased serum collagen I in AF patients and atrial fibrosis in the left atrium, further suggesting a profibrotic role for SPP1 in this context.

A recent study suggests that SPP1 plays a crucial role in immune regulation [23]. Elevated SPP1 expression has been shown to influence CD4^+^ T cell differentiation, promoting the Th17 phenotype while inhibiting Treg development, primarily through stabilization of HIF-1α via inhibition of its degradation [21]. These effects have been noted in conditions such as chronic pulmonary inflammation and hepatic steatosis [24, 25]. Our findings of a reduced Treg/Th17 ratio in AF patients, alongside elevated SPP1 levels, support the hypothesis that SPP1 contributes to immune dysregulation in AF.

An imbalanced Treg/Th17 ratio has been previously associated with inflammatory cardiovascular diseases such as atherosclerosis and viral myocarditis [26, 27]. Treg cells typically exert protective effects by suppressing immune activation through IL-10 secretion, whereas Th17 cells promote inflammation and tissue injury [28, 29]. Our results are consistent with this paradigm, as both AF patients and AF rats demonstrated reduced anti-inflammatory markers alongside increased pro-inflammatory markers.

However, this study also has several limitations. First, the sample size was limited, and a causal relationship between SPP1 and the Treg/Th17 imbalance has not been established. Second, the specific molecular mechanisms by which SPP1 modulates T cell differentiation in AF remain to be elucidated. Future mechanistic studies are needed to elucidate the role of SPP1 in AF pathogenesis and to evaluate its potential as a therapeutic target.

## 5. Conclusion

In conclusion, our study demonstrates that serum SPP1 levels are inversely correlated with the Treg/Th17 ratio in NVAF. Elevated SPP1 levels in AF are associated with both immune imbalance and fibrotic remodeling, suggesting a dual role in disease progression. These findings provide novel insights into the immunoinflammatory mechanisms underlying AF, highlighting SPP1 as a promising biomarker and potential therapeutic target.

## Availability of Data and Materials

The datasets used and analysed during the current study are available from the corresponding author on reasonable request.
